# Exploring the role of the atherogenic index of plasma as a mediator between body roundness Index and cardiovascular events in older adults: a NHANES-based study

**DOI:** 10.3389/fcvm.2025.1506603

**Published:** 2025-05-14

**Authors:** Kangming Li, Shizhong Chen, Yanan Hu, Chunmei Qi

**Affiliations:** ^1^ Department of Cardiology, The Second Affiliated Hospital of Xuzhou Medical University, Xuzhou, China; ^2^ Graduate School, Xuzhou Medical University, Xuzhou, Jiangsu, China

**Keywords:** body roundness index, atherogenic index of plasma, older adults, cardiovascular diseases, national health and nutrition examination survey, mediation analysis

## Abstract

**Background:**

The rising incidence of cardiovascular diseases (CVD) in the elderly highlights the need for effective preventive strategies. Recent studies suggest that obesity, through metabolic factors, contributes to the development of CVD. This study aims to explore how body roundness index (BRI) levels affect the occurrence of CVD using data from the National Health and Nutrition Examination Survey (NHANES) (2003–2016), to better understand the role of obesity in CVD prevention and management.

**Methods:**

The study analyzed data from 3,584 NHANES participants over seven cycles (2003–2016), dividing them into three groups (T1, T2, T3) based on BRI values. Univariate and multivariate regression analyses were used to assess the association between BRI and atherogenic index of plasma (AIP) levels with the occurrence of CVD. The mediating effect of AIP on BRI and CVD was also analyzed.

**Results:**

Compared to the lowest tertile of BRI, participants with higher BRI levels had a higher proportion of females, smokers, drinkers, and individuals with lower educational attainment. Poverty-income ratio (PIR) and AIP levels were significantly higher, and the prevalence of CVD was also higher. BRI and AIP were both independent risk factors for CVD, with AIP having a significant mediating effect between BRI and CVD.

**Conclusion:**

BRI levels significantly impact the occurrence of CVD through AIP mediation.

## Introduction

Cardiovascular diseases (CVD) is one of the major global public health issues. In 2020, it was estimated that approximately 19 million people worldwide died from CVD, an 18.7% increase from 2010 (1). Age is closely associated with CVD. A study conducted in 2021 identified three critical age milestones for cardiovascular health: 25–45 years, 45–65 years, and over 65 years. It was noted that individuals over 65 are particularly vulnerable to cardiovascular risks, which subsequently increases healthcare demand (2). As populations age, improving cardiovascular health in individuals aged 65 and over has become a key research focus in public health. Numerous studies have identified this age group as high-risk for myocardial infarction, stroke, and heart failure (3, 4). After experiencing adverse cardiovascular events, patients may suffer a severe decline in their quality of life, affecting daily living, social activities, and mental health. Thus, effectively assessing and predicting the risk of cardiovascular events in the elderly is crucial for formulating preventive and treatment strategies.

Research has shown that central obesity is an independent risk factor for CVD (5). While traditional body mass index (BMI) is commonly used to assess obesity, it fails to accurately reflect fat distribution (6), whereas the Body Roundness Index (BRI), which incorporates height and waist circumference, provides a more precise evaluation of abdominal fat. It is calculated using the following formula: BRI = 364.2 − 365.5 × √(1 − [Waist circumference (cm)/2*π*]2/[0.5 × Height (cm) ]) (7). Compared to BMI and waist circumference alone, BRI has shown a stronger correlation with cardiovascular risk (7–9). BRI allows for a more effective assessment of the risks associated with body fat distribution in older adults, which plays a key role in cardiovascular health (10). In parallel, the Atherogenic Index of Plasma (AIP), which is derived from the ratio of triglyceride (TG) to high-density lipoprotein cholesterol (HDL-C), has gained recognition as an important marker of lipid metabolism. AIP is calculated as: AIP = lg[TG (mmol/L)/HDL-C (mmol/L)] (11). where TG is the triglyceride level (mg/dl) and HDLC is the high-density lipoprotein cholesterol level (mg/dl). Triglycerides are a type of fat found in the blood, and their levels are closely associated with the presence of small, dense, atherogenic particles (12). These triglyceride-rich lipoproteins play a significant role in the development of atherosclerosis. This index reflects the partial esterification rate of HDL-C and the balance between protective and atherogenic lipoproteins. A growing body of evidence supports its association with cardiovascular disease, showing that AIP provides superior predictive value for CVD outcomes compared to traditional lipid parameters (13–15). Although BRI and AIP are independently associated with cardiovascular risk, the potential mediating role of AIP between BRI and cardiovascular outcomes in the elderly has not been fully explored.

**Figure 1:**
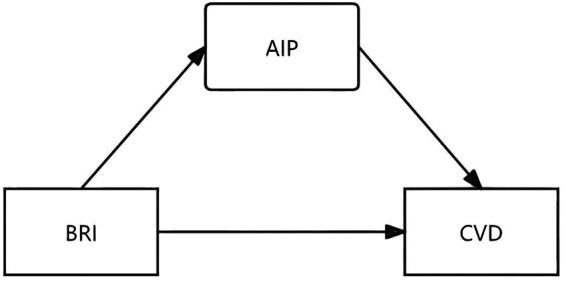
This study hypothesized that AIP has an intermediate effect between BRI and CVD. BRI: Body roundness index; AIP: Atherogenic index of plasma; CVD: Cardiovascular diseases.

The novelty of this study lies in its first comprehensive analysis of the impact of the BRI on CVD risk in elderly individuals using large-scale data from the NHANES database and further uncovers the mediating role of the AIP in this relationship. The study hypothesize that AIP, by reflecting lipid metabolism abnormalities, amplifies the association between BRI and CVD, thereby elucidating the underlying mechanisms linking central obesity to cardiovascular health (Figure 1). This study provides new insights into early risk assessment and personalized management strategies for CVD in older adults, addressing gaps in understanding the synergistic effects of BRI and AIP.

### Study population

The Centers for Disease Control and Prevention (CDC) conducts the National Health and Nutrition Examination Survey (NHANES) to evaluate the health and dietary habits of the U.S. population. It employs a detailed, stratified multistage sampling approach to ensure the data is representative. Each cycle of NHANES collects extensive information, such as demographic data, physical measurements, lab results, and food intake (16). Full details on the datasets are available on http://www.cdc.gov/nchs/nhanes.html.

**Figure 2:**
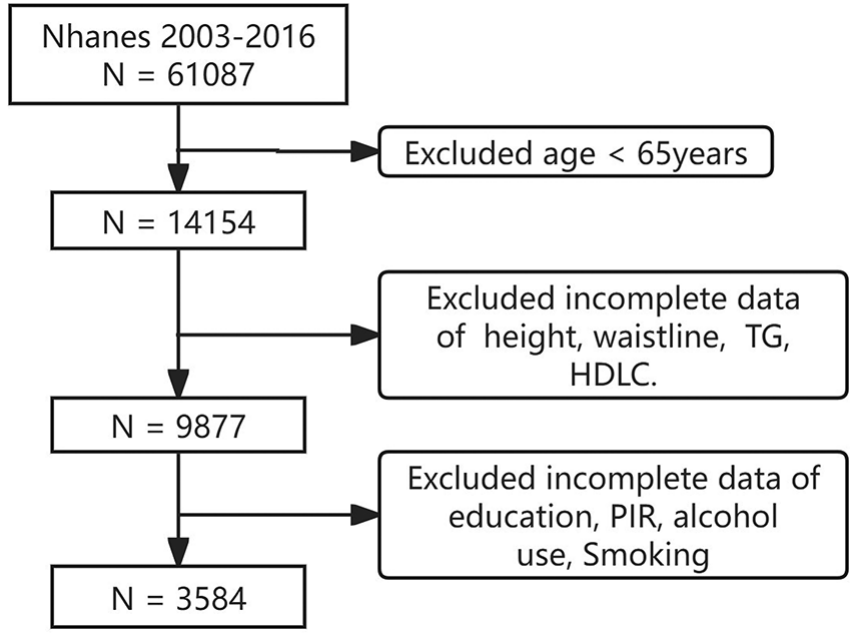
Study flowchart.

The study screened and analyzed data from 2003 to 2016. Exclusion criteria for this study (1) individuals younger than 65 years of age, (2) individuals without waistline, height, TG and HDL-C, and (3) individuals lacking necessary covariate records, such as age, gender, alcohol consumption, smoking status, race, and education level. A total of 3,584 participants were included in the study from 2003 to 2016 (Figure 2).

### Variables

Sex, Race, Education level, Height, Waist circumference, TG, HDL-C. Smoking status was determined by the question: “Have you ever smoked at least 100 cigarettes in your lifetime?” (SMQ020). Those who smoked fewer than 100 cigarettes were classified as never smokers (17). Alcohol consumption was assessed using the question: “In any given year, have you had at least 12 alcoholic drinks?” (ALQ101) (18). Individuals responding “Yes” to this question were classified as alcohol consumers. Cardiovascular disease (CVD) diagnoses were self-reported through a questionnaire for heart failure (MCQ160b), coronary heart disease (MCQ160c), angina (MCQ160d), heart attack (MCQ160e), and stroke (MCQ160f). These conditions were collectively defined as cardiovascular diseases. This study used in the data is available at http://www.cdc.gov/nchs/nhanes.html.

### Statistical analysis

Statistical analyses were performed using R 4.3.1. Categorical data were described as *n* (%), and continuous variables were described as mean ± standard deviation (x ± s). Univariate and multivariate regression analyses were conducted to assess correlations, and a mediation model was constructed to estimate the direct, indirect, and total effects of pathways. Adjusted 95% confidence intervals (CI) were calculated. An effect was considered statistically significant if the 95% CI did not include 0, with a significance level of *P* < 0.05.

## Results

### Participant characteristics

The baseline characteristics of participants across different BRI tertiles are shown. BRI values were 9.45 ± 1.43 in T1, 12.85 ± 0.87 in T2, and 17.84 ± 3.07 in T3. Compared to the lower BRI groups, participants with higher BRI were more likely to be female, smokers, drinkers, and had lower education levels (*P* < 0.05). Additionally, those in higher tertiles had significantly higher levels of TG, AIP and lower HDLC levels, with a higher prevalence of a history of cardiovascular events (*P* < 0.05) (Table 1).

**Table 1:** Characteristics of the study participants (*N* = 3,584).

Characteristics	Tertiles of BRI			*P*
	T1(*N* = 1,195)	T2(*N* = 1,195)	T3(*N* = 1,194)	
BRI	9.45 ± 1.43	12.85 ± 0.87	17.84 ± 3.07	<0.001
Age, years	73.41 ± 5.51	73.21 ± 5.37	72.79 ± 5.18	0.016
Sex (%)				
Female	526 (44.0%)	534 (44.7%)	734 (61.5%)	<0.001
Male	669 (56.0%)	661 (55.3%)	460 (38.5%)	
Race (%)				
Mexican American	67 (5.6%)	128 (10.7%)	157 (13.1%)	<0.001
Other Hispanic	93 (7.8%)	149 (12.5%)	173 (14.5%)	
Non-Hispanic White	698 (58.4%)	660 (55.2%)	615 (51.5%)	
Non-Hispanic Black	310 (25.9%)	247 (20.7%)	211 (17.7%)	
Other/multiracial	27 (2.3%)	11 (0.9%)	38 (3.2%)	
Education level, n(%)				
Less Than 9th Grade	146 (12.2%)	193 (16.2%)	223 (18.7%)	<0.001
9–11th Grade	162 (13.6%)	160 (13.4%)	174 (14.6%)	
High School Grad/GED	255 (21.4%)	282 (23.6%)	288 (24.1%)	
Some College or AA degree	344 (28.8%)	319 (26.7%)	332 (27.8%)	
College Graduate or above	286 (24.0%)	241 (20.2%)	176 (14.8%)	
PIR	2.65 ± 1.53	2.49 ± 1.54	2.17 ± 1.46	<0.001
Smoke.group (%)				
No	534 (44.7%)	567 (47.4%)	612 (51.3%)	0.005
Yes	661 (55.3%)	628 (52.6%)	582 (48.7%)	
alq.group (%)				
No	318 (26.6%)	370 (31.0%)	475 (39.8%)	<0.001
Yes	877 (73.4%)	825 (69.0%)	719 (60.2%)	
BMI, kg/m^2^	23.79 ± 2.79	28.37 ± 2.55	34.85 ± 4.91	<0.001
Waist circumference, cm	89.79 ± 8.33	102.30 ± 6.56	116.41 ± 10.50	<0.001
Height, cm	167.02 ± 9.72	165.61 ± 9.89	162.47 ± 9.73	<0.001
CVD (%)				
NO	896 (75.0%)	890 (74.5%)	837 (70.1%)	0.012
YES	299 (25.0%)	305 (25.5%)	357 (29.9%)	
TG, mmol/L	1.36 ± 0.85	1.71 ± 1.07	1.99 ± 1.10	<0.001
HDL-C, mmol/L	1.57 ± 0.48	1.38 ± 0.41	1.30 ± 0.36	<0.001
AIP	−0.25 ± 0.71	0.10 ± 0.72	0.33 ± 0.68	<0.001

PIR, ratio of family income to poverty; BRI, body roundness index; BMI, body mass index; CVD, cardiovascular diseases; TG, triglyceride; HDL-C, high-density lipoprotein cholesterol; AIP, atherogenic index of plasma.

### BRI and cardiovascular disease

Factors that could influence cardiovascular events from baseline data were included in the regression equation to control for confounders. Model 1 was unadjusted for confounding factors, while Model 2 adjusted for sex, race, and education level. Model 3 further adjusted for poverty index, smoking, and alcohol history (Table 2). BRI was identified as a risk factor for CVD (OR = 1.005, 95% CI: 1.002–1.009, *P* < 0.001).

**Table 2:** Association between BRI and CVD.

Model	Variable	OR	95%CI	*P*
Model 1	BRI	1.007	1.003 – 1.011	<0.001
Model 2	BRI	1.008	1.005 – 1.012	<0.001
Model 3	BRI	1.005	1.002 – 1.009	<0.001

Model 1 represents the unadjusted analysis; Model 2 is adjusted for sex, race, and education level; Model 3 is adjusted for sex, race, education level, PIR, smoking history, and alcohol consumption history. PIR, ratio of family income to poverty; BRI, body roundness index; OR, odds ratio; 95%CI, 95% confidence interval.

### AIP and cardiovascular disease

Factors that could influence cardiovascular events from baseline data were included in the regression equation to control for confounders. Model 1 was unadjusted for confounding factors, while Model 2 adjusted for sex, race, and education level. Model 3 further adjusted for poverty index, smoking, alcohol consumption history, and BRI (Table 3). AIP was found to be an independent risk factor for CVD (OR = 1.027, 95% CI: 1.005–1.050, *P* < 0.001).

**Table 3:** Association between AIP and CVD.

Model	Variable	OR	95%CI	*P*
Model 1	AIP	1.063	1.042 – 1.030	<0.001
Model 2	AIP	1.056	1.035 – 1.077	<0.001
Model 3	AIP	1.027	1.004 – 1.050	<0.001

Model 1 represents the unadjusted analysis; Model 2 is adjusted for sex, race, and education level; Model 3 is adjusted for sex, race, education level, PIR, BRI, smoking history, and alcohol consumption history. PIR, ratio of family income to poverty; BRI, body roundness index; AIP, atherogenic index of plasma; OR, odds ratio; 95%CI, 95% confidence interval.

### Mediation analysis

**Figure 3:**
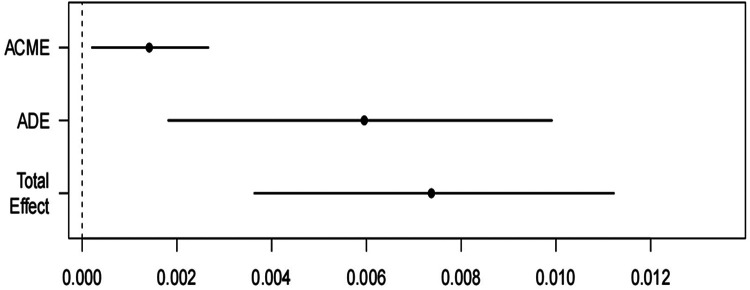
Relationship model among the study variables. ACME: Average Causal Mediation Effect; ADE Average Direct Effect.

To validate the robustness of the study findings, demographic variables were adjusted in the mediation analysis. The results showed a significant direct effect of BRI on the incidence of cardiovascular disease (*P* = 0.004). Additionally, AIP, acting as a mediator, demonstrated a significant indirect effect (*P* = 0.034), further strengthening the association between BRI and cardiovascular disease (Figure 3).

## Discussion

CVD is closely related to the quality of life of patients, which not only damages the physical function and sleep quality of patients, but also aggravates the feeling of loneliness of patients, and brings fatigue and lack of sleep to the family members (19–21). With advances in diagnosis and treatment technology, the prognosis of cardiovascular disease has been sufficiently improved, but according to one study, the average annual costs of cardiovascular disease in the U.S. in 2017–2018 were estimated at $378 billion, and predicted that the pressure of cardiovascular disease on the social health care system will continue to increase in 2025–2050. This is mainly driven by the ageing of the global population (22–25). At the same time, studies have pointed out that elderly people over 65 years old are generally faced with cardiovascular risk and are at high risk of CVD (2). Therefore, it is particularly important to formulate scientific and reasonable prevention and treatment strategies for this kind of elderly population to improve people's happiness index and reduce social medical burden.

Obesity is a well-established independent risk factor for CVD and has traditionally been assessed using body mass index (BMI). However, recent research in 2024 highlights the limitations of BMI, as it does not accurately reflect visceral fat thickness or differentiate between muscle and fat mass. Abdominal fat, particularly visceral fat surrounding key organs such as the liver, has been identified as a stronger predictor of CVD than BMI (26). The BRI, which incorporates both height and waist circumference, provides a more precise assessment of abdominal fat and has shown a stronger correlation with CVD incidence. Researchs have also shown that BRI exhibits superior discriminatory power in assessing metabolic disease risk, particularly when accounting for gender differences, compared to traditional measures such as BMI and waist circumference (7–9, 27). Additionally, the relationship between obesity and CVD is partially mediated by the triglyceride-glucose (TyG) index, indicating that metabolic factors play a role in obesity-related CVD risk (28). Lipid metabolism is a crucial component of cardiovascular risk, and the AIP, a composite lipid marker, offers a more comprehensive evaluation of lipid status, AIP has shown superior predictive ability for CVD compared to individual lipid parameters and is also associated with BRI, suggesting a potential interrelationship between these two indices in the context of cardiovascular health (13–15, 29). Both BRI and AIP are independent markers of metabolic disturbances, with higher levels of BRI reflecting increased central obesity and a greater risk of CVD. Similarly, elevated AIP values, which represent an imbalance in lipid metabolism, have been linked to an increased risk of atherosclerosis and cardiovascular events. However, we recognize that HDL-C is a highly heterogeneous molecule, and its concentration may not fully reflect the patient's lipid profile, especially if influenced by oxidative stress and inflammation, which can modify HDL-C and its functionality (30, 31). Despite these complexities, previous studies have established AIP, calculated as the ratio of TG to HDL-C, as an important marker for cardiovascular risk (32, 33). Given that both BRI and AIP are implicated in the pathophysiology of cardiovascular diseases, it appears that AIP may serve as an intermediary, bridging the association between BRI and CVD risk.

Using data from the NHANES database, this study provides robust evidence that the AIP plays a significant biological role in the pathway linking the BRI to CVD, as supported by epidemiological analysis. Notably, both BRI and AIP consistently demonstrated strong associations with CVD across various model adjustments. Mediation analysis, controlling for demographic variables, revealed a significant direct effect of BRI on the incidence of CVD, while AIP exhibited a notable indirect effect, further reinforcing the relationship between BRI and CVD. These findings underscore the potential of incorporating BRI and AIP into clinical practice for more precise cardiovascular risk stratification, particularly in populations at higher risk of metabolic and cardiovascular disorders. Early identification and intervention based on these indices may improve preventative strategies and optimize patient outcomes. Future research should focus on longitudinal studies to validate the causal relationships observed in this cross-sectional analysis. Additionally, exploring the impact of lifestyle interventions, such as targeted dietary and physical activity programs, on modulating BRI and AIP could provide actionable insights for reducing cardiovascular risk. Integrating these indices into personalized risk prediction models may further enhance their clinical utility and contribute to the development of precision medicine in cardiovascular care.

### Strength

Firstly, it is the first to explore the relationship between the BRI and the incidence of CVD mediated by the AIP. Secondly, robust statistical methods were utilized, with thorough adjustments for confounding variables, to ensure the reliability of the findings. Thirdly, the analysis was based on data derived from a large-scale population database, which adhered to rigorous quality control protocols.

### Limitation

This study has several limitations that should be considered when interpreting the results. First, the cross-sectional design limits our ability to establish causal relationships between the BRI and CVD. Additionally, the study only involved a single measurement of BRI and the Atherogenic Index of Plasma (AIP), without longitudinal follow-up to assess how changes in these indices influence CVD outcomes over time. Moreover, important clinical variables such as the use of medications (e.g., antihypertensive agents, statins, anti-hyperglycemic medications, and anti-platelets) were not included in the analysis, which may affect the relationship between BRI and CVD. Furthermore, the study did not gather data on the prevalence of comorbidities such as diabetes, arterial hypertension, hypercholesterolemia, obstructive sleep apnea syndrome (OSAS), migraine, or atrial fibrillation—all of which are known to increase CVD risk, particularly in a population in primary prevention. Additionally, we did not account for physical activity levels, which are known to significantly influence cardiovascular risk. The absence of these variables could impact the results and their generalizability. Future studies should incorporate these factors, and we suggest further exploration of the role of medication use, comorbid conditions, and lifestyle factors such as physical activity in modulating the relationship between BRI and CVD.

## Conclusion

In this NHANES-based study involving 3,584 participants, we found that the Body Roundness Index (BRI) is significantly associated with the occurrence of cardiovascular disease (CVD), and this relationship is partially mediated by the Atherogenic Index of Plasma (AIP). Our findings suggest that a reduction in BRI, particularly in older adults, may contribute to a lower risk of CVD. Mediation analysis further supported that AIP plays a significant role in linking BRI to CVD occurrence. These results underscore the importance of managing lifestyle factors, such as body fat distribution, to mitigate the risk of cardiovascular diseases in older adults.

## Data Availability

The datasets presented in this study can be found in online repositories. The names of the repository/repositories and accession number(s) can be found below: http://www.cdc.gov/nchs/nhanes.html.

## References

[1] Martin SS Aday AW Almarzooq ZI Anderson CAM Arora P Avery CL 2024 heart disease and stroke statistics: a report of US and global data from the American Heart Association. Circulation. (2024) 149(8):e347–913. 10.1161/CIR.0000000000001209 38264914 PMC12146881

[2] Jakob J Stalder O Kali T Pruvot E Pletcher MJ Rana JS The coronary artery risk development in young adults (CARDIA) study. Am J Med. (2022) 135(7):871. 10.1016/j.amjmed.2022.01.057 35245494

[3] Weissler EH Wang Y Gales JM Feldman DN Arya S Secemsky EA Cardiovascular and limb events following endovascular revascularization among patients ≥65 years old: an American College of Cardiology PVI registry analysis. J Am Heart Assoc. (2022) 11(12):e024279. 10.1161/JAHA.121.024279 35723018 PMC9238644

[4] Marquina C Talic S Vargas-Torres S Petrova M Abushanab D Owen A Future burden of cardiovascular disease in Australia: impact on health and economic outcomes between 2020 and 2029. Eur J Prev Cardiol. (2022) 29(8):1212–19. 10.1093/eurjpc/zwab001 33686414

[5] Ntimana CB Seakamela KP Mashaba RG Maimela E. Determinants of central obesity in children and adolescents and associated complications in South Africa: a systematic review. Front Public Health. (2024) 12:1324855–55. 10.3389/fpubh.2024.1324855 38716247 PMC11075369

[6] Müller MJ Lagerpusch M Enderle J Schautz B Heller M Bosy-Westphal A. Beyond the body mass index: tracking body composition in the pathogenesis of obesity and the metabolic syndrome. Obes Rev. (2012) 13(Suppl 2):6–13. 10.1111/j.1467-789X.2012.01033.x 23107255

[7] Thomas DM Bredlau C Bosy-Westphal A Mueller M Shen W Gallagher D Relationships between body roundness with body fat and visceral adipose tissue emerging from a new geometrical model. Obesity. (2013) 21(11):2264–71. 10.1002/oby.20408 23519954 PMC3692604

[8] Zhang X Ma N Lin Q Chen K Zheng F Wu J Body roundness index and all-cause mortality among US adults. JAMA Netw Open. (2024) 7(6):e2415051–e51. 10.1001/jamanetworkopen.2024.15051 38837158 PMC11154161

[9] Cai X Song S Hu J Zhu Q Yang W Hong J Body roundness index improves the predictive value of cardiovascular disease risk in hypertensive patients with obstructive sleep apnea: a cohort study. Clin Exp Hypertens. (2023) 45(1):2259132. 10.1080/10641963.2023.2259132 37805984

[10] Franek E Pais P Basile J Nicolay C Raha S Hickey A General versus central adiposity as risk factors for cardiovascular-related outcomes in a high-risk population with type 2 diabetes: a *post hoc* analysis of the REWIND trial. Cardiovasc Diabetol. (2023) 22(1):52–52. 10.1186/s12933-023-01757-z 36899386 PMC9999507

[11] Min Q Wu Z Yao J Wang S Duan L Liu S Association between atherogenic index of plasma control level and incident cardiovascular disease in middle-aged and elderly Chinese individuals with abnormal glucose metabolism. Cardiovasc Diabetol. (2024) 23(1):54–54. 10.1186/s12933-024-02144-y 38331798 PMC10854096

[12] Cole J Couture P Tremblay AJ Sniderman AD. Variance in the composition and number of VLDL and LDL particles with increasing triglyceride or increasing ApoB concentrations. J Clin Lipidol. (2025) 19(1):72–82. 10.1016/j.jacl.2024.09.009 39532567

[13] Šebeková K Gurecká R Csongová M Koborová I Celec P. Association of atherogenic index of plasma with cardiometabolic risk factors and markers in lean 14-to-20-year-old individuals: a cross-sectional study. Children (Basel). (2023) 10(7):1144. 10.3390/children10071144 37508640 PMC10378605

[14] Lioy B Webb RJ Amirabdollahian F. The association between the atherogenic index of plasma and cardiometabolic risk factors: a review. Healthcare (Basel). (2023) 11(7):966. 10.3390/healthcare11070966 37046893 PMC10094587

[15] Tamosiunas A Luksiene D Kranciukaite-Butylkiniene D Radisauskas R Sopagiene D Bobak M. Predictive importance of the visceral adiposity index and atherogenic index of plasma of all-cause and cardiovascular disease mortality in middle-aged and elderly Lithuanian population. Front Public Health. (2023) 11:1150563. 10.3389/fpubh.2023.1150563 36992890 PMC10040644

[16] Liang H Si W Li L Yang K. Association between body roundness index and osteoarthritis: a cross-sectional analysis of NHANES 2011–2018. Front Nutr. (2024) 11:1501722–22. 10.3389/fnut.2024.1501722 39545042 PMC11560466

[17] Wang X Mukherjee B Park SK. Associations of cumulative exposure to heavy metal mixtures with obesity and its comorbidities among U.S. adults in NHANES 2003–2014. Environ Int. (2018) 121(P1):683–94. 10.1016/j.envint.2018.09.035 30316184 PMC6268112

[18] Chen HL Wu C Cao L Wang R Zhang TY He Z. The association between the neutrophil-to-lymphocyte ratio and type 2 diabetes mellitus: a cross-sectional study. BMC Endocr Disord. (2024) 24(1):107. 10.1186/s12902-024-01637-x 38982402 PMC11232124

[19] Schopfer DW Beatty AL Meyer CS Whooley MA. Longitudinal association between angina pectoris and quality of life. Am J Cardiol. (2022) 164:1–6. 10.1016/j.amjcard.2021.10.037 34838288

[20] Zhang J Chai X Ye Y Zhao Q Fan X. Association between sleep and quality of life in heart failure patient-caregiver dyads and mediation of fatigue: an actor-partner interdependence mediation model. J Adv Nurs. (2022) 78(8):2436–47. 10.1111/jan.15174 35133026

[21] Li H Zheng D Li Z Wu Z Feng W Cao X Association of depressive symptoms with incident cardiovascular diseases in middle-aged and older Chinese adults. JAMA Netw Open. (2019) 2(12):e1916591. 10.1001/jamanetworkopen.2019.16591 31800066 PMC6902756

[22] Xue Q Wu S He X Huang Y Liu Y Yan T Trends in cardiovascular health metrics and associations with long-term mortality among US adults with coronary heart disease. Nutr Metab Cardiovasc Dis. (2024) 34(8):1932–41. 10.1016/j.numecd.2024.03.031 38755082

[23] Koehler F Koehler J Bramlage P Vettorazzi E Wegscheider K Lezius S Impact of telemedical management on hospitalization and mortality in heart failure patients with diabetes: a *post-hoc* subgroup analysis of the TIM-HF2 trial. Cardiovasc Diabetol. (2024) 23(1):198–98. 10.1186/s12933-024-02285-0 38867198 PMC11170842

[24] Chong B Jayabaskaran J Jauhari SM Chan SP Goh R Kueh MT Global burden of cardiovascular diseases: projections from 2025 to 2050. Eur J Prev Cardiol. (2024):zwae281. 10.1093/eurjpc/zwae281 39270739

[25] Zhang Q Wang A Xu Q Xia X Tian X Zhang Y Efficacy and safety of Ginkgo diterpene lactone meglumine in acute ischemic stroke: a randomized clinical trial. JAMA Netw Open. (2023) 6(8):e2328828–e28. 10.1001/jamanetworkopen.2023.28828 37578791 PMC10425831

[26] Busetto L Dicker D Frühbeck G Halford JCG Sbraccia P Yumuk V A new framework for the diagnosis, staging and management of obesity in adults. Nat Med. (2024) 30(9):2395–99. 10.1038/s41591-024-03095-3 38969880

[27] Zhao W Tong J Li J Cao Y. Relationship between body roundness index and risk of type 2 diabetes in Japanese men and women: a reanalysis of a cohort study. Int J Endocrinol. (2021) 2021:4535983. 10.1155/2021/4535983 35003255 PMC8731295

[28] Tian X Chen S Wang P Xu Q Zhang Y Luo Y Insulin resistance mediates obesity-related risk of cardiovascular disease: a prospective cohort study. Cardiovasc Diabetol. (2022) 21(1):289–89. 10.1186/s12933-022-01729-9 36564775 PMC9789633

[29] Genshan Z Haokun Z Jie F Yufeng Z. Atherogenic index of plasma as a mediator in the association between body roundness index and depression: insights from NHANES 2005–2018. Lipids Health Dis. (2024) 23(1):183–83. 10.1186/s12944-024-02177-y 38867232 PMC11167922

[30] Bucurica S Nancoff AS Dutu M Mititelu MR Gaman LE Ioniță-Radu F Exploring the relationship between lipid profile, inflammatory state and 25-OH vitamin D serum levels in hospitalized patients. Biomedicines. (2024) 12(8):1686–86. 10.3390/biomedicines12081686 39200151 PMC11351771

[31] Hany M Demerdash HM Abouelnasr AA Agayby AS Ibrahim M Arida RE Relationship between weight loss, changes in serum hs-CRP levels and apo A-1 lipoprotein, and high-density lipoprotein-cholesterol ratios as predictors for improved cardiovascular risk factors after laparoscopic sleeve gastrectomy. Obes Surg. (2024) 34(9):1–11. 10.1007/s11695-024-07441-9 39141186 PMC11349864

[32] Liu Z Zhang L Wang L Li K Fan F Jia J The predictive value of cumulative atherogenic index of plasma (AIP) for cardiovascular outcomes: a prospective community-based cohort study. Cardiovasc Diabetol. (2024) 23(1):264–64. 10.1186/s12933-024-02350-8 39026310 PMC11264486

[33] Liu Y Feng X Yang J Zhai G Zhang B Guo Q The relation between atherogenic index of plasma and cardiovascular outcomes in prediabetic individuals with unstable angina pectoris. BMC Endocr Disord. (2023) 23(1):187–87. 10.1186/s12902-023-01443-x 37653411 PMC10469417

